# Differential Effects of 17β-Estradiol, Its Metabolites, and Cadmium on Cytotoxicity and Redox-Related Pathways in Doxorubicin-Sensitive and -Resistant Breast Cancer Cell Lines

**DOI:** 10.3390/ph19071001

**Published:** 2026-06-28

**Authors:** Ewa Sawicka, Katarzyna Zdybel, Martyna Wolniak, Agnieszka Piwowar

**Affiliations:** 1 Department of Toxicology, Faculty of Pharmacy, Wroclaw Medical University, Borowska 211, 50-556 Wroclaw, Poland; agnieszka.piwowar@umw.edu.pl; 2 Students’ Scientific Society, Department of Toxicology, Faculty of Pharmacy, Wroclaw Medical University, 50-556 Wroclaw, Poland; zdybel.katarzyna@gmail.com (K.Z.); martynawolniak30@gmail.com (M.W.)

**Keywords:** breast cancer cell line, cadmium chloride, 17β-estradiol, 2-methoxyestradiol, 4-hydroxyestradiol, SOD1, GST, GST-pi, interaction

## Abstract

**Background**: Breast cancer is the most common malignancy among women and a leading cause of cancer-related deaths globally. Its development involves hormonal, genetic, environmental and inflammatory factors. Among environmental contributors, cadmium (Cd^2+^), a metalloestrogen known to induce redox imbalance, as well as estrogen metabolites, may exert divergent biological effects. **Methods**: This study investigated the effects of 17β-estradiol (E2) and its metabolites—2-methoxyestradiol (2-MeOE2) and 4-hydroxyestradiol (4-OHE2)—administered alone or in combination with CdCl_2_, on estrogen receptor–-positive MCF-7 breast cancer cells and their doxorubicin-resistant cells (MCF-7/DOX). We evaluated cytotoxicity, interaction profiles (synergism/antagonism), and redox-related enzymes associated with drug resistance, including superoxide dismutase 1 (SOD1) and glutathione S-transferase pi (GST-pi). There are no known examples of these types of interactions, especially those involving estrogen metabolites with opposing biological activities—anticancer 2-MeOE2 and procarcinogenic—4-OHE2 in combination with cadmium. Cell viability was assessed after 48 h exposure to individual and combined treatments of CdCl_2_. Interaction types (synergism/antagonism) were determined via the combination index method. Antioxidative enzymes were evaluated by quantitative and immunocytochemical analysis of SOD1, GST and GST-pi expression. **Results**: All tested compounds reduced cell viability in a concentration-dependent manner, with CdCl_2_ showing the highest cytotoxicity. MCF-7 cell lines were generally more sensitive to CdCl_2_, E2, and 2-MeOE2, whereas MCF-7/DOX cell lines exhibited greater sensitivity to 4-OHE2. Combination studies revealed predominantly antagonistic interactions, particularly for CdCl_2_ + 2-MeOE2, suggesting a protective redox-modulating effect of this metabolite. Resistant cells consistently displayed higher SOD1 activity and GST-pi expression, indicating enhanced adaptive responses to oxidative stress. **Conclusions**: Our study underscores the importance of concentration-dependent interactions between environmental Cd^2+^ and pathways regulated by 17β-estradiol and its metabolites, particularly in the context of cytotoxicity and redox imbalance relevant to breast cancer progression and therapy resistance.

## 1. Introduction

Breast cancer is the most frequently diagnosed malignancy among women and remains a leading cause of cancer-related death worldwide. According to recent global cancer statistics, over 2.26 million new cases were diagnosed in 2020 [1,2]. The development of breast cancer is multifactorial and involves genetic predisposition, hormonal regulation and environmental exposures. Environmental xenobiotics that induce oxidative stress may play a significant role in the initiation and progression of carcinogenic processes in hormone-dependent cancers [3,4,5].

Among them, metalloestrogens—such as cadmium—are of particular concern due to their ability to activate estrogen receptors and induce oxidative stress-dependent responses. Cadmium (Cd) is a highly toxic, non-essential transition metal and a significant environmental pollutant [6]. In biological systems, cadmium occurs primarily as a divalent cation (Cd^2+^), which is the predominant biologically active form, whereas Cd^+^ is unstable and occurs only rarely. Cd^2+^ acts as a strong metalloestrogen, capable of activating estrogen receptor alpha (ERα) and related receptors, thereby modulating estrogen-regulated pathways in hormone-dependent cancers. Through its ability to induce redox imbalance, Cd^2+^ can influence oxidative stress responses and adaptive cellular pathways, which may contribute to cytotoxicity and differential responses in both drug-sensitive and doxorubicin-resistant breast cancer cells [7]. Experimental studies have demonstrated that cadmium can activate estrogen receptors and mimic certain biological effects of 17β-estradiol (E2), thereby stimulating proliferation of estrogen receptor-positive breast cancer cells such as the MCF-7 line. In addition, cadmium can induce oxidative stress and epigenetic alterations, all of which have been implicated in carcinogenesis [8,9]. Epidemiological studies further suggest an association between cadmium exposure and increased breast cancer risk, and higher cadmium concentrations have been reported in breast tumor tissues compared with adjacent normal tissues [10,11,12].

In addition to environmental factors, endogenous estrogens and their metabolites may also contribute to breast cancer development. Estrogen metabolism leads to the formation of several biologically active metabolites, including 2-hydroxyestradiol (2-OHE2), 2-methoxyestradiol (2-MeOE2), and 4-hydroxyestradiol (4-OHE2) [13,14]. These metabolites differ significantly in their biological activity. For example, 2-MeOE2 has been reported to exhibit anti-proliferative, antioxidant, and anti-inflammatory properties, whereas catechol estrogens such as 4-OHE2 may promote oxidative stress, generate reactive oxygen species, and induce DNA damage, thereby contributing to carcinogenic processes [15].

Both estrogens and cadmium are known to influence cellular redox homeostasis and antioxidant defense systems. Enzymes such as superoxide dismutase (SOD), glutathione S-transferase (GST), and its isoenzyme GSTpi (GST-pi) play key roles in maintaining redox balance, regulating inflammation-associated stress responses, and cellular adaptation to toxic insults [16,17].

Alterations in the activity or expression of these enzymes have been associated with tumor progression as well as resistance to anticancer therapy. Increased SOD activity has been observed in breast cancer tissues and in estrogen receptor-positive breast cancer cells, suggesting a role in adaptive responses to oxidative stress. Similarly, elevated GST expression has been linked to enhanced detoxification capacity and the development of multidrug resistance in cancer cells [18,19]. GST-pi is the most functionally and clinically relevant isoform in breast cancer, which justifies its selection for this study [20].

Chemotherapy resistance remains a major challenge in breast cancer treatment, and doxorubicin is one of the commonly used drugs in breast cancer therapy. Mechanisms underlying resistance to doxorubicin are frequently associated with alterations in oxidative stress responses and detoxification pathways, including enzymes such as SOD and GST-pi. Therefore, investigating these processes in drug-resistant cells may provide important insight into mechanisms that contribute to the survival and adaptation of cancer cells under therapeutic pressure [21,22].

Although both cadmium and estrogens are known to influence oxidative stress-related pathways, their combined effects on antioxidant defense mechanisms in breast cancer cells remain insufficiently characterized, particularly in the context of chemotherapy resistance. Moreover, little is known about how cadmium interacts with biologically distinct estrogen metabolites that may exert either pro-carcinogenic or anticancer effects [23].

To the best of our knowledge, the combined effects of cadmium and biologically distinct estrogen metabolites on oxidative stress-related pathways in both doxorubicin-sensitive and doxorubicin-resistant breast cancer cells have not been previously investigated.

Therefore, the aim of this study was to investigate the effects of E2 and its metabolites, 2-MeOE2 and 4-OHE2, and their interactions with CdCl_2_ in estrogen receptor-positive breast cancer cells. Specifically, we evaluated their effects on cell viability, the type of interaction between these compounds, oxidative stress assessed by superoxide dismutase (SOD) activity, and the expression of the detoxification enzyme glutathione S-transferase pi (GST-pi). To further explore the potential relevance of these mechanisms to chemotherapy resistance, the experiments were performed in both doxorubicin-sensitive (MCF-7) and doxorubicin-resistant (MCF-7/DOX) breast cancer cell lines. This comparative approach allowed us to assess whether cadmium and estrogen metabolites differentially modulate oxidative stress responses and detoxification pathways in drug-sensitive and drug-resistant breast cancer cells.

## 2. Results

### 2.1. Assessment of Cytotoxicity of Individual Compounds on MCF-7 and MCF-7/DOX Cell Lines

**Figure 1:**
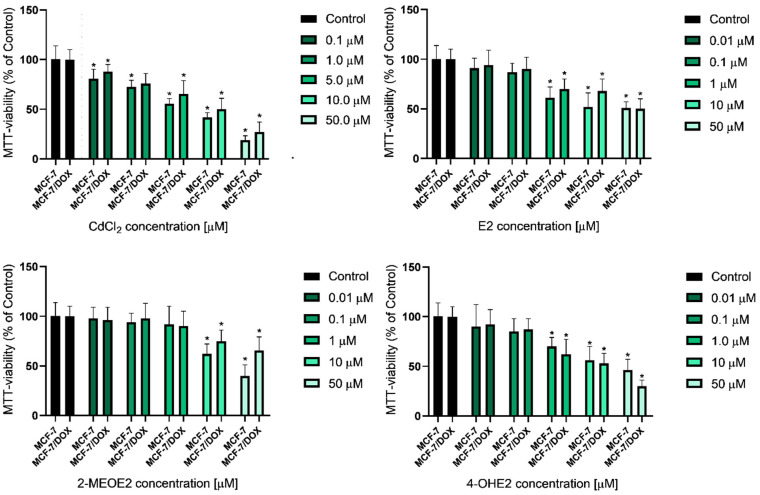
Comparison of the viability of two cell lines: MCF-7 and MCF-7/DOX after 48 h exposure to CdCl_2_, E2, 2-MeOE2, and 4-OHE2 determined by MTT test; significance in comparison to control (* *p* < 0.05).

To determine the cytotoxic profiles of the tested compounds and to optimize their concentrations for subsequent combination studies, cell viability assays were performed on MCF-7 and MCF-7/DOX breast cancer cells following exposure to individual agents. The tested concentrations were as follows: E2, 2-MeOE2 and 4-OHE2 at 0.01, 0.1, 1.0, 10.0, and 50.0 µM; cadmium chloride (CdCl_2_) at 0.1, 1.0, 5.0, 10.0, and 50.0 µM. The MTT assay was performed after 48 h of incubation of cells. Figure 1 presents the viability of both cell lines following exposure to cadmium ions, E2, 2-MeOE2 and 4-OHE2 in the above concentrations.

The results demonstrated a statistically significant decrease in cell viability of both MCF-7 and MCF-7/DOX lines. Cytotoxic effects were most pronounced at higher concentrations—specifically, at 1, 10, and 50 μM for E2 and 4-OHE2, and at 10 and 50 μM for 2-MeOE2. The most substantial reduction in cell viability was observed following exposure to cadmium ions, which exhibited cytotoxic effects at concentrations as low as 1 μM. At this concentration, a statistically significant decrease in viability was noted compared to the untreated control (*p* < 0.05). Moreover, the observed reduction in cell viability was concentration-dependent.

When comparing the responses of the two cell lines, MCF-7 cells appeared more sensitive to Cd, E2, and 2-MeOE2 exposure, as evidenced by lower viability compared to MCF-7/DOX cells. This suggests that the doxorubicin-resistant MCF-7/DOX cells exhibit reduced sensitivity to these compounds. This difference in sensitivity was particularly evident at concentrations above 1 μM for cadmium and from 10 μM for E2 and 2-MeOE2. In contrast, exposure to 4-OHE2 resulted in a reversed trend: a significantly greater reduction in viability was observed in MCF-7/DOX cells compared to the parental MCF-7 line (*p* < 0.05) (Figure 1). Based on the calculated IC_50_ values for the tested compounds, cadmium exhibited the highest cytotoxicity toward MCF-7 cells (IC_50_ = 4.26 µM), whereas 4-OHE2 showed the greatest cytotoxic effect on MCF-7/DOX cells (IC_50_ = 6.74 µM) (Table 1).

### 2.2. Assessment of Cytotoxicity of Simultaneous Action of CdCl_2_ with E2, 2-MeOE2, and 4-OHE2- on MCF-7 and MCF-7/DOX Cell Lines–Interaction Study

To determine appropriate concentrations for the combined cadmium and estrogens exposure study, we considered data from experiments involving individual compounds, previous studies conducted on ovarian cancer cell lines, and the relevant literature. Based on this analysis, a range of estrogen concentrations was selected to evaluate potential interactions: for E2, 2-MeOE2, and 4-OHE2, the concentrations used were 0.01, 0.1, 1, 10, and 50 μM. For cadmium (CdCl_2_), we selected two concentrations: the lowest (1 μM) and the highest (50 μM) that significantly reduced the viability of both MCF-7 and MCF-7/DOX cell lines compared to the control (*p* < 0.05).

#### 2.2.1. Combined Effects of E2 and Cadmium

**Figure 2:**
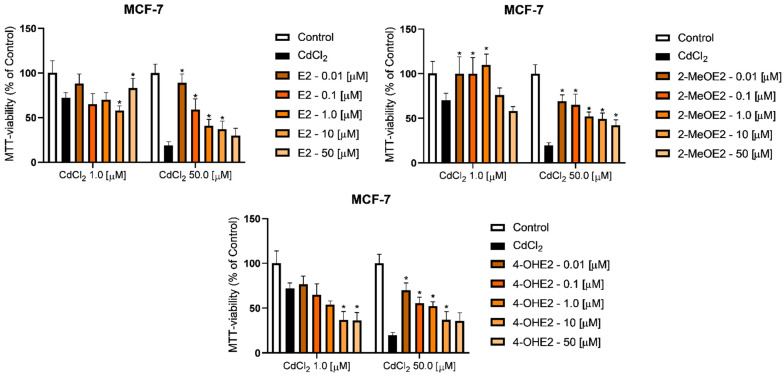
Viability of the MCF-7 cell line after combined exposure for 48 h to E2 or 2-MeOE2 or 4-OHE2 with CdCl_2_ determined by an MTT test in comparison to CdCl_2_ alone at concentrations of 1 µM and 50 µM (* *p* < 0.05).

**Figure 3:**
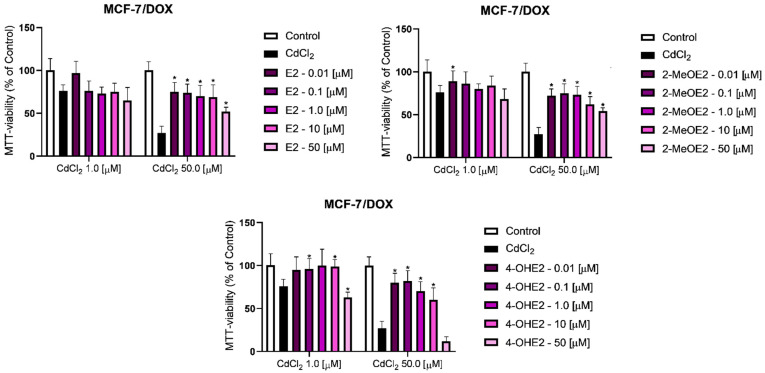
Viability of the MCF-7/DOX cell line after combined exposure for 48 h to E2 or 2-MeOE2 or 4-OHE2 with CdCl_2_ determined by an MTT test in comparison to CdCl_2_ alone at concentrations of 1 µM and 50 µM (* *p* < 0.05).

In the co-exposure experiments, the MCF-7 cell line was more sensitive to the cadmium–estrogen mixtures, particularly at the highest cadmium concentration (50 μM). A statistically significant decrease in cell viability was observed following combined exposure, compared to cadmium alone. The combined treatment of 50 μM Cd and 50 μM E2 resulted in a pronounced reduction in cell viability, indicating increased sensitivity of MCF-7 cells to this mixture (Figure 2). The interaction type between Cd and E2 in the MCF-7 cell line, calculated using the combination index method, is summarized in Table 2. The nature of the interaction was concentration-dependent. At the lower cadmium concentration (1 μM), synergy was observed for E2 at 10 μM. At the higher cadmium concentration (50 μM), synergistic effects were noted for E2 concentrations of 1 and 10 μM. All other Cd–E2 combinations showed antagonistic interactions, suggesting a potential protective effect of E2 against cadmium toxicity in MCF-7 cells. In contrast, the MCF-7/DOX cell line exhibited lower sensitivity to combined cadmium–estrogen exposure, as evidenced by significantly higher cell viability compared to MCF-7 under the same treatment conditions (Figure 3). Synergistic interactions were observed at the lower cadmium concentration (1 μM) for E2 at 0.1 and 1 μM. However, the overall interaction profile was similar in both cell lines, with antagonism being the predominant interaction type, further supporting the notion of a protective effect of E2 against cadmium-induced cytotoxicity. The interaction type between Cd and E2 the MCF-7/DOX cell line, calculated using the combination index method, is summarized in Table 3.

#### 2.2.2. Combined Effects of 2-MeOE2 and Cadmium

Evaluation of the combined effects of 2-MeOE2 and cadmium revealed no substantial differences in the responses between the chemotherapy-sensitive (MCF-7) and -resistant (MCF-7/DOX) breast cancer cell lines. Both cell lines exhibited comparable sensitivity to the 2-MeOE2–Cd mixtures. The overall pattern of changes, as illustrated in Figure 1 and Figure 2, was similar in both lines. However, in MCF-7 cells—which were initially more sensitive to the tested compounds—2-MeOE2, a metabolite with reported anticancer properties, exerted a statistically significant protective effect by increasing cell viability across nearly the entire concentration range when co-exposed with either 1 μM or 50 μM of Cd. Across all tested concentrations, interactions between 2-MeOE2 and Cd were predominantly antagonistic, suggesting a protective role of this E2 metabolite in counteracting cadmium-induced cytotoxicity.

#### 2.2.3. Combined Effects of 4-OHE2 and Cadmium

In contrast, co-exposure to cadmium and 4-OHE2, a metabolite with potential carcinogenic activity, elicited a more diverse response between the two cell lines. The MCF-7 cells, known to be more sensitive, exhibited a greater decrease in viability particularly at the lower cadmium concentration (1 μM). Conversely, MCF-7/DOX cells responded differently: for most tested concentrations, combined exposure resulted in higher overall cell viability compared with treatment with 1 μM Cd alone (Figure 3).

However, when exposed to 50 μM Cd, both cell lines showed a similar trend: a statistically significant increase in cell viability compared to cadmium alone, indicating a possible antagonistic interaction. Notably, in the chemoresistant MCF-7/DOX cells, a striking observation was made: co-exposure to the highest concentrations of both compounds (50 μM 4-OHE2 and 50 μM CdCl_2_) drastically reduced cell viability to 12%, a significantly greater reduction than that caused by cadmium alone (24%). The interaction at this concentration was classified as synergistic (Table 4).

Nevertheless, the majority of interactions between 4-OHE2 and cadmium across the concentration range were antagonistic, again suggesting a potential modulatory or protective effect of the estrogen metabolite, depending on concentration and cell line.

Based on the calculated IC50 values, cadmium chloride (CdCl_2_) exhibited cytotoxic effects in both MCF-7 and MCF-7/DOX breast cancer cell lines, with MCF-7 cells being more sensitive (IC_50_ = 4.26 µM vs. 9.74 µM for MCF-7/DOX). Co-treatment with E2, 2-MeOE2, or 4-OHE2 can change CdCl_2_ cytotoxicity in a concentration– and cell line-dependent manner. In MCF-7 cells, high CdCl_2_ concentrations, particularly with E2 or 4-OHE2, led to markedly enhanced cytotoxicity (IC_50_ = 1.04 µM and 0.55 µM, respectively).

In MCF-7/DOX cells, CdCl_2_ sensitivity was generally lower, consistent with multidrug-resistant phenotypes. 2-MeOE2 maintained a protective effect even at high CdCl_2_ concentrations, whereas 4-OHE2 increased cytotoxicity, although this was less pronounced than in MCF-7 cells.

Treatment with cadmium and estrogen metabolites led to a significant decrease in cell viability in both MCF-7 and MCF-7/DOX cell lines. This effect was more pronounced in MCF-7 cells, whereas MCF-7/DOX cells exhibited partial resistance. These observations are consistent with the known effects of cadmium and 4-hydroxyestradiol on oxidative stress and redox imbalance, which can compromise cell survival [14,24].

### 2.3. Assessment of SOD1 Expression Following Exposure to Individual Compounds in Two Cell Lines: MCF-7 and MCF-7/DOX—Immunocytochemical Staining

The analysis of SOD1 expression following exposure to cadmium and estrogenic compounds revealed distinct patterns of oxidative response between the MCF-7 and MCF-7/DOX cell lines, as well as concentration-dependent effects for each compound. Under control conditions, baseline SOD1 expression was moderate in both cell lines, although slightly higher in MCF-7/DOX (++ vs. + in MCF-7), suggesting an inherently enhanced oxidative defense mechanism in the chemoresistant cells.

In MCF-7 cells, cadmium exposure induced a mild to moderate increase in SOD1 expression, with intensity rising from + at 0.1 μM to +/++ at 10 μM. In contrast, MCF-7/DOX cells showed a markedly stronger response to the same cadmium concentrations, with ++ at 0.1 μM and ++/+++ at 10 μM, indicating a more robust expression of antioxidant defenses under cadmium-induced oxidative stress.

The response to E2 was notably different between the two lines. In MCF-7 cells, E2 at 0.1 μM led to moderate SOD1 expression (++), which paradoxically decreased to + at the higher concentration (10 μM). This may reflect a concentration-dependent dual effect of E2, potentially shifting from antioxidant to pro-oxidant properties at higher concentrations. In contrast, MCF-7/DOX cells demonstrated consistently elevated SOD1 expression (++ at 0.1 μM, ++/+++ at 10 μM), suggesting enhanced sensitivity or altered redox regulation in the chemoresistant phenotype.

Both cell lines responded similarly to 2-MeOE2. At 0.1 μM, SOD1 expression was moderately elevated (++), but decreased to + at 10 μM in both MCF-7 and MCF-7/DOX cells. This pattern indicates a concentration-dependent decline in SOD induction, potentially linked to the known pro-apoptotic and antiangiogenic effects of 2-MeOE2 at higher concentrations.

Among all tested compounds, 4-OHE2 induced the strongest SOD response. In MCF-7 cells, the intensity ranged from +/++ at 0.1 μM to ++/+++ at 10 μM. Similarly, MCF-7/DOX cells showed increased SOD induction at both concentrations (++ and ++/+++), which may suggest that this metabolite, with potential pro-oxidant and carcinogenic properties, may activate protective pathways against oxidative stress in both cell types. The semi-quantitative grading of SOD1 immunocytochemical staining after exposure to individual and combined compounds in both examined cell lines is shown in Table 5 and Table 6.

### 2.4. Assessment of SOD1 Expression Following Exposure to Combined Effect of Compounds in Two Cell Line: MCF-7 and MCF-7/DOX—Immunocytochemical Staining

The combined exposure of CdCl_2_ with different estrogens revealed compound-specific and concentration-dependent modulation of SOD1 activity in both MCF-7 and MCF-7/DOX breast cancer cell lines. Overall, the chemoresistant MCF-7/DOX cells showed a somewhat stronger SOD1 response to the combined treatments, which may be related to their increased ability to adapt to oxidative stress.

#### 2.4.1. Combined Effects of E2 and Cadmium

In MCF-7 cells, co-exposure to low concentrations of E2 (0.1 µM) and cadmium (0.1 or 10 µM) resulted in moderate SOD1 activation (both ++). At a higher E2 concentration (10 µM), however, SOD1 activity remained at a similar level (++ at 0.1 µM Cd) or declined (+ at 10 µM Cd), suggesting a potential concentration-dependent dampening of the antioxidant response.

In contrast, MCF-7/DOX cells showed stronger SOD1 responses to the same combinations. Notably, treatment with low-concentration E2 (0.1 µM) and Cd resulted in SOD1 intensities of +++ and ++/+++, respectively, indicating a synergistic activation of antioxidant defense mechanisms. At higher E2 (10 µM), SOD1 activity remained stable (++), regardless of Cd concentration.

#### 2.4.2. Combined Effects of 2-MeOE2 and Cadmium

2-MeOE2 co-treatment with cadmium induced a particularly strong SOD1 response in both cell lines. In MCF-7 cells, low-concentration 2-MeOE2 (0.1 µM) in combination with either 0.1 or 10 µM Cd significantly increased SOD1 activity (+++ for both). A similar trend was observed in MCF-7/DOX cells (++ at 0.1 µM Cd and +++ at 10 µM Cd), suggesting that 2-MeOE2 potently enhances the cellular antioxidant response under oxidative stress.

When a higher concentration of 2-MeOE2 (10 µM) was used, SOD1 activation was still elevated in both lines, although it was slightly lower in MCF-7 (++ for both Cd concentrations), while MCF-7/DOX maintained a strong response (++/+++ and +++). These results showed the effect of 2-MeOE2, particularly under conditions of oxidative stress.

#### 2.4.3. Combined Effects of 4-OHE2 and Cadmium

4-OHE2, a known pro-oxidant metabolite of estradiol, produced a variable SOD1 response depending on the concentration and cell line. In MCF-7 cells, combined exposure to 0.1 µM 4-OHE2 and cadmium resulted in moderate to strong SOD1 activation (+/++ at 0.1 µM Cd; +++ at 10 µM Cd). However, a higher 4-OHE2 concentration (10 µM) did not further increase activity, and SOD1 levels remained at +/++ for both Cd concentrations.

**Figure 4:**
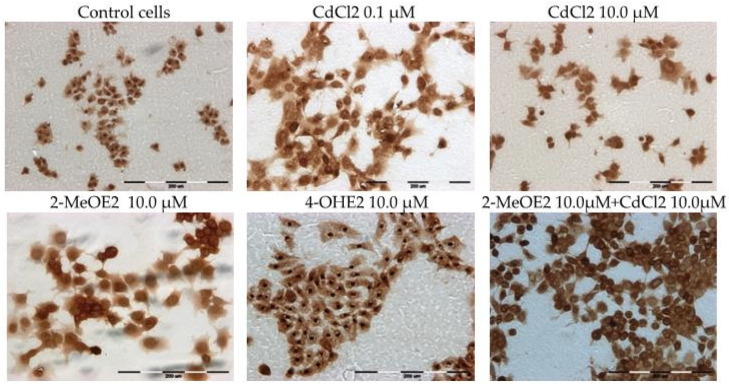
Immunoassayed reaction: exemplary expression results of SOD1 in the MCF-7 cell, showing both exposure to the single compounds and exposure to the combined effect of estrogens and CdCl_2_.

**Figure 5:**
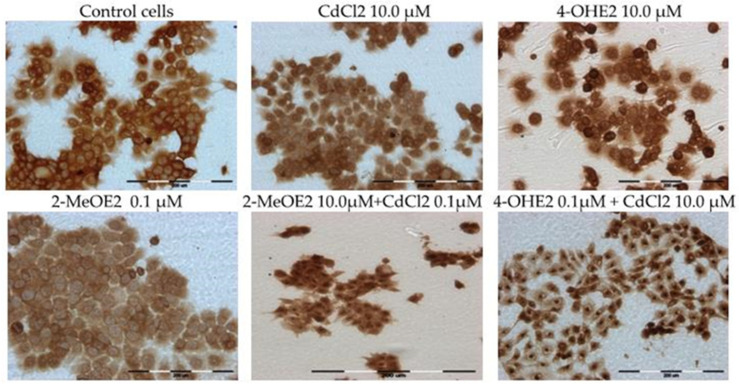
Immunoassayed reaction: exemplary expression results of SOD1 in the MCF-7/DOX cell, showing both exposure to the single compounds and exposure to the combined effects of estrogens and CdCl_2_.

MCF-7/DOX cells again showed a more pronounced response: co-treatment with low-concentration 4-OHE2 yielded ++/+++ and +++ SOD1 levels, while higher 4-OHE2 concentrations maintained strong activity (++ and ++/+++). This indicates that even pro-oxidant estrogens can trigger protective antioxidant mechanisms in resistant cells. Under control conditions, baseline SOD1 activity was higher in MCF-7/DOX (++) than in MCF-7 (+), further supporting the concept of enhanced antioxidant readiness in drug-resistant cells. All combined treatments—particularly those involving 2-MeOE2 and 4-OHE2—produced greater SOD1 induction than controls, with the strongest effects seen in MCF-7/DOX (Figure 4 and Figure 5).

### 2.5. Assessment of GST-pi Expression Following Exposure to Individual Compounds in Two Cell Line: MCF-7 and MCF-7/DOX—Immunocytochemical Staining

Immunocytochemical analysis suggests that the expression of GST-pi can be changed by the tested compounds in both MCF-7 and MCF-7/DOX breast cancer cell lines. The staining intensity, used as a semi-quantitative indicator of enzyme expression, was evaluated in response to CdCl_2_, E2, 2-MeOE2 and 4-OHE2, at two concentrations (0.1 µM and 10.0 µM), and compared to untreated controls.

Under control conditions, both MCF-7 and MCF-7/DOX cells exhibited low but detectable GST-π expression, marked by weak staining (+). This may suggest the presence of a basic detoxification capacity, which likely supports the maintenance of redox homeostasis in both cell lines.

Exposure to CdCl_2_ resulted in a concentration-dependent increase in GST-pi expression in both cell lines. In MCF-7 cells, staining intensity increased from + at 0.1 µM to ++/++ at 10 µM. The MCF-7/DOX cells showed an even more pronounced response, with GST-pi expression increasing from ++ at 0.1 µM to ++/+++ at 10 µM. These findings suggest that cadmium is a potent inducer of GST-pi, particularly in the drug-resistant MCF-7/DOX line. This enhanced response may reflect an adaptive mechanism associated with chemoresistance, as GST-pi contributes to xenobiotic detoxification and neutralization of reactive oxygen species (ROS), both of which are relevant to cadmium toxicity.

Treatment with E2 also led to an increase in GST-pi expression, although the response varied between cell lines. In MCF-7, expression increased from +/++ at 0.1 µM to +++ at 10 µM, indicating a strong hormone-induced activation of the enzyme. In contrast, MCF-7/DOX showed a less consistent pattern, with staining intensities of ++ at 0.1 µM and +/++ at 10 µM. This differential response may result from altered estrogen receptor (ER) signaling or transcriptional regulation in drug-resistant cells. The strong response in MCF-7 is consistent with the hormone sensitivity of this ER-positive line.

2-MeOE2 induced a marked increase in GST-pi expression in MCF-7 cells, with staining intensities of ++ at 0.1 µM and ++/+++ at 10 µM. In MCF-7/DOX cells, the response was weaker and did not change significantly with concentration, remaining at +/++. These results suggest that 2-MeOE2 may act as an inducer of GST-pi in sensitive breast cancer cells, although its effectiveness appears to be lower in drug-resistant cells.

Among the tested compounds, 4-OHE2 induced the least variation in GST-pi expression. In MCF-7 cells, both concentrations caused mild to moderate staining (+/++), with no apparent concentration dependency. In MCF-7/DOX, the response was slightly higher at 0.1 µM (++), but remained unchanged at 10 µM. The modest effect of 4-OHE2 suggests that this metabolite has limited capacity to upregulate GST-pi, at least under the tested conditions. This is notable considering the reported genotoxicity of catechol estrogens, indicating that GST-pi regulation in this context may be influenced by additional oxidative or DNA damage signals. The semi-quantitative grading of GST-pi immunocytochemical staining after exposure to individual and combined compounds in both examined cell lines is shown in Table 7 and Table 8.

### 2.6. Assessment of GST-pi Expression Following Exposure to Combined Effect of Compounds in Two Cell Line: MCF-7 and MCF-7/DOX—Immunocytochemical Staining

The immunocytochemical analysis of GST-pi expression following co-exposure to cadmium chloride and estrogens (E2, 2-MeOE2, and 4-OHE2) revealed compound- and cell line-dependent responses. Expression levels were assessed based on the intensity of staining, compared to control conditions.

Baseline expression of GST-pi was weak in MCF-7 cells (+), whereas in the drug-resistant MCF-7/DOX line, moderate staining (++) was observed. This difference may confirm a higher constitutive level of GST-pi in MCF-7/DOX, consistent with its multidrug-resistant phenotype.

#### 2.6.1. Combined Effects of E2 and Cadmium

In MCF-7 cells, co-treatment with E2 and CdCl_2_ (at both low and high concentrations) resulted in moderate to strong expression (++ to +++), suggesting an additive or slightly synergistic effect on GST-pi upregulation. E2 (10.0 µM) + CdCl_2_ (0.1 µM) induced the strongest response (+++), suggesting that estrogen at a higher concentration significantly contributes to GST-pi induction even in the presence of low cadmium levels. In contrast, in MCF-7/DOX cells co-treatment with E2 (0.1 µM) and CdCl_2_ induced strong (+++) or moderate (++) responses, depending on concentration. The strongest reaction (+++) occurred at low concentrations of both agents, suggesting hypersensitivity or non-linear interactions in the resistant line. These results indicate that although MCF-7 responds more consistently to increased E2 concentration in combination with CdCl_2_, the MCF-7/DOX line may exhibit different regulation of detoxification enzymes.

#### 2.6.2. Combined Effects of 2-MeOE2 and Cadmium

Combined treatment with 2-MeOE2 and CdCl_2_ led to moderate (++), and in some cases strong (++/+++) GST-pi expression in both cell lines. In MCF-7, all combinations yielded consistent moderate expression (++), with no significant concentration dependency. In MCF-7/DOX, the combination of 2-MeOE2 10 µM + CdCl_2_ 10 µM induced the strongest response (+++), indicating concentration-dependent enhancement in the resistant line. This suggests that 2-MeOE2 maintains its GST-pi-inducing potential even in the presence of cadmium, particularly in chemoresistant cells, where redox stress signaling may be upregulated.

#### 2.6.3. Combined Effects of 4-OHE2 and Cadmium

**Figure 6:**
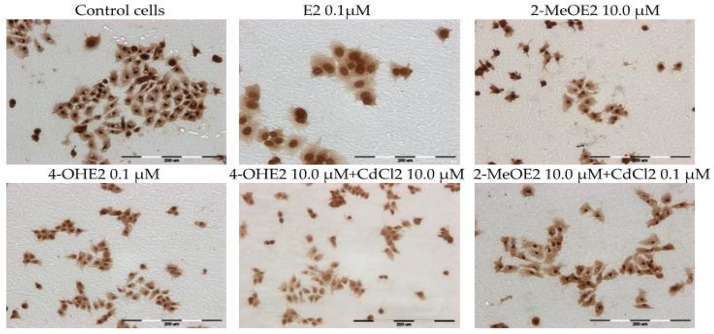
Immunoassayed reaction: exemplary expression results of GST-pi in the MCF-7 cell, showing both exposure to the single compounds and exposure to the combined effect of estrogens and CdCl_2_.

**Figure 7:**
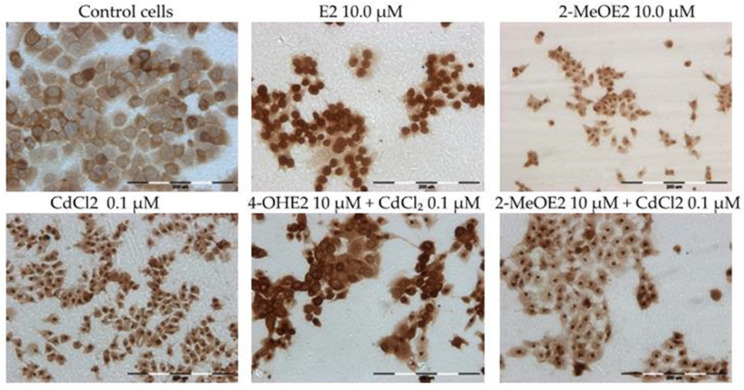
Immunoassayed reaction: exemplary expression results of GST-pi in the MCF-7/DOX cell, showing both exposure to the single compounds and exposure to the combined effects of estrogens and CdCl_2_.

Among the tested combinations, 4-OHE2 co-treatment showed the most variable and cell line-dependent effects. In MCF-7, GST-pi expression remained moderate (++), regardless of the concentration of either compound. In MCF-7/DOX, a more complex pattern emerged: low concentrations induced only mild-to-moderate responses (+/++), while 4-OHE2 10 µM + CdCl_2_ 0.1–10 µM significantly elevated GST-pi expression (+++ and ++/+++). This may reflect an enhanced pro-oxidative response in MCF-7/DOX cells to catechol estrogens like 4-OHE2, particularly under conditions of additional cadmium-induced stress (Figure 6 and Figure 7).

### 2.7. Assessment of SOD1 Activity Following to Single and Combined Exposure to CdCl_2_ and Estrogens in MCF-7 and MCF-7/DOX Cell Lines Using the RANSOD Spectrophotometric Assay

#### 2.7.1. Effect of Single Compounds on SOD1 Activity

Exposure of MCF-7 and MCF-7/DOX cells to single compounds resulted in a concentration-dependent modulation of SOD1 activity compared with untreated controls. In parental MCF-7 cells, CdCl_2_ induced a significant increase in SOD1 activity at both concentrations, with a more pronounced effect observed at 10 µM (22.8 ± 1.4 U/mg protein vs. 12.4 ± 0.8 U/mg protein in control). Estrogenic compounds also elevated SOD1 activity, although the magnitude of the response varied depending on the compound and concentration. E2 induced only a slight increase at 0.1 µM, whereas a more evident elevation was observed at 10 µM. The strongest induction among all estrogens was detected after exposure to 4-OHE2, which increased SOD1 activity up to 30.6 ± 2.1 U/mg protein at 10 µM. Similarly, 2-MeOE2 significantly enhanced SOD1 activity in a concentration-dependent manner. In the doxorubicin-resistant MCF-7/DOX cell line, basal SOD1 activity was markedly higher compared to parental MCF-7 cells. Exposure to CdCl_2_ further increased SOD1 activity, reaching 31.7 ± 1.9 U/mg protein at 10 µM. All tested estrogens also induced SOD1 activity in MCF-7/DOX cells, with a stronger response than in MCF-7 cells. In particular, 4-OHE2 and 2-MeOE2 caused the most pronounced elevation of SOD1 activity, reaching 41.2 ± 2.5 and 35.9 ± 2.0 U/mg protein, respectively, at the highest concentration. Overall, among the tested compounds, 4-OHE2 exerted the strongest stimulatory effect on SOD1 activity in both cell lines, while CdCl_2_ consistently acted as a potent inducer of antioxidant response. Moreover, MCF-7/DOX cells exhibited consistently higher basal SOD1 activity compared with parental MCF-7 cells, indicating an enhanced antioxidant capacity associated with the resistant phenotype (Table 9).

#### 2.7.2. Effect of Combined Action of Compounds on SOD1 Activity

Co-exposure of MCF-7 and MCF-7/DOX cells to CdCl_2_ and estrogens (E2, 2-MeOE2, and 4-OHE2) resulted in a more pronounced increase in SOD1 activity compared with single compound treatments. In MCF-7 cells, combined exposure induced a concentration-dependent increase in SOD1 activity that exceeded the values observed after individual treatment with either CdCl_2_ or estrogens alone. The most pronounced effect was observed for 4-OHE2 in combination with CdCl_2_ at 10 µM, where SOD1 activity reached 43.5 ± 2.6 U/mg protein, representing a clear increase compared with 4-OHE2 alone (30.6 ± 2.1 U/mg protein) and CdCl_2_ alone (22.8 ± 1.4 U/mg protein). A similar pattern was observed in MCF-7/DOX cells, where combined treatment induced a stronger increase in SOD1 activity than single exposures. The highest enzymatic activity was detected following co-treatment with 4-OHE2 and CdCl_2_ at 10 µM (56.4 ± 3.5 U/mg protein), which also exceeded the effects of 4-OHE2 alone (41.2 ± 2.5 U/mg protein) and CdCl_2_ alone (31.7 ± 1.9 U/mg protein). In summary, the results indicate that simultaneous exposure to cadmium and estrogens enhances the cellular antioxidant response to a greater extent than individual treatments. This effect was particularly evident for 4-OHE2 and in the resistant MCF-7/DOX cell line, which exhibited a higher adaptive antioxidant capacity (Table 10).

### 2.8. Assessment of GST Activity Following Single and Combined Exposure to CdCl_2_ and Estrogens in MCF-7 and MCF-7/DOX Cell Lines Using a CDNB-Based Spectrophotometric Assay

#### 2.8.1. Effect of Single Compounds on GST Activity

Exposure of MCF-7 and MCF-7/DOX cells to Cd, estradiol, and its metabolites resulted in a concentration-dependent increase in GST activity compared with the respective controls. Basal GST activity was higher in MCF-7/DOX cells than in MCF-7 cells. Among the tested compounds, 4-OHE2 induced the strongest increase in GST activity, followed by 2-MeOE2, whereas E2 exerted a weaker effect also in MCF-7 in comparison to the MCF-7/DOX line. Cadmium at 10 µM significantly enhanced GST activity in both cell lines but especially in MCF-7/DOX. The highest GST activity was observed in MCF-7/DOX cells treated with 10 µM 4-OHE2. Almost all tested compounds caused an increase in GST activity compared to the appropriate controls (Table 11).

#### 2.8.2. Effect of Combined Action of Compounds on GST Activity

Combined exposure to CdCl_2_ and estrogens or their metabolites resulted in a pronounced increase in GST activity in both MCF-7 and MCF-7/DOX cells compared with the respective control groups as well as with treatments involving lower concentrations of the tested compounds. The magnitude of GST induction depended on both the type of estrogenic compound and its concentration, indicating a dose-dependent activation of the glutathione-dependent detoxification system.

In MCF-7 cells, GST activity increased from the control value of 42.8 ± 2.6 nmol/min/mg protein to 82.7 ± 4.9 and 95.4 ± 5.7 nmol/min/mg protein following combined treatment with CdCl_2_ (10.0 µM) and E2 at concentrations of 0.1 and 10.0 µM, respectively. Exposure to 2-MeOE2 induced a stronger response, reaching 101.8 ± 6.1 and 119.7 ± 7.1 nmol/min/mg protein, whereas the highest GST activities were observed after treatment with 4-OHE2, which increased enzyme activity to 114.8 ± 6.8 and 136.2 ± 8.1 nmol/min/mg protein at 0.1 and 10.0 µM, respectively.

A similar but more pronounced pattern was observed in MCF-7/DOX cells. Basal GST activity in this cell line (68.5 ± 4.1 nmol/min/mg protein) was substantially higher than in parental MCF-7 cells. Co-exposure to CdCl_2_ and E2 increased GST activity to 114.2 ± 6.8 and 128.6 ± 7.5 nmol/min/mg protein, while treatment with 2-MeOE2 elevated GST activity to 137.2 ± 8.0 and 158.4 ± 9.3 nmol/min/mg protein. The strongest stimulation was recorded following combined exposure to CdCl_2_ and 4-OHE2, resulting in GST activities of 149.5 ± 8.8 and 182.9 ± 10.7 nmol/min/mg protein at the lower and higher estrogen concentrations, respectively.

## 3. Discussion

Xenoestrogens, or hormonally active environmental compounds, are increasingly recognized as modulators of estrogen receptor activity as well as contributors to oxidative and inflammation-related cellular responses. Among these, metalloestrogens—such as cadmium, cobalt, copper, nickel, and chromium—are of particular concern due to their ability to activate estrogen receptors in the absence of endogenous ligands such as estradiol. This receptor activation is associated with mechanisms involved in the pathogenesis of estrogen-dependent cancers, including breast cancer [25,26,27].

Cadmium is a well-known, widespread environmental carcinogen, requiring careful assessment of its biological effects. However, the combined effects of cadmium and endogenous estrogens, particularly their metabolites, on breast cancer development and inflammation-related pathways remain poorly understood. Cd^2+^ acts as a strong metalloestrogen, capable of activating estrogen receptor alpha (ERα) and related receptors, thereby modulating estrogen-regulated pathways in hormone-dependent cancers. In experimental studies, cadmium was used in its divalent form (Cd^2+^, oxidation state +2), which is the biologically active and relevant valence state responsible for its endocrine-disrupting and cellular effects [7]. Metabolic biotransformation of E2 can generate both protective and carcinogenic metabolites, potentially altering the cellular response to xenoestrogens through modulation of redox balance and stress signaling [14,15]. This raises a key question: Does cadmium exposure modify the physiological or pathological effects of estrogens on breast tissue, particularly in the context of cytotoxicity and redox-related pathways?

Given that oxidative stress and impaired antioxidant defense mechanisms play a key role in breast cancer progression and are closely linked to inflammatory signaling, we expanded our analysis to include the assessment of superoxide dismutase (SOD) expression using immunocytochemical methods. This approach allowed us to investigate how combined exposure to estrogens and cadmium affects cellular viability and redox homeostasis.

The present study aimed to explore the interaction between cadmium and estrogenic compounds (E2, 2-MeOE2, and 4-OHE2) in two breast cancer cell lines: hormone-sensitive MCF-7 and doxorubicin-resistant MCF-7/DOX. The investigation focused on cytotoxic effects as well as the responses of the antioxidant enzyme SOD1 and detoxification enzyme GST-pi, both involved in redox regulation and estrogenic signaling, to elucidate the oxidative and estrogenic pathways underlying these interactions. The obtained results appear interesting and may open a new avenue of research aimed at better understanding these interactions and developing potential protective strategies.

### 3.1. Differential Sensitivity of MCF-7 and MCF-7/DOX to Individual Agents

In vitro studies were conducted in two models: single exposure and combined exposure. This is crucial considering the cumulative exposure of the human body to a range of environmental toxins. The cell lines used are cell models used in breast cancer research. The MCF-7 is the original line, while MCF-7/DOX was bred to achieve drug resistance. Initial cytotoxicity testing revealed that both cell lines responded to the tested compounds in a concentration-dependent manner, with MCF-7 cells displaying overall greater sensitivity. Notably, cadmium demonstrated cytotoxicity at low micromolar concentrations, consistent with previous reports on its potent pro-oxidant, endocrine-disrupting, and inflammation-promoting properties [28,29,30]. MCF-7/DOX cells exhibited a relatively higher resistance to Cd, E2 and 2-MeOE2, which is likely related to their altered redox balance and enhanced defense mechanisms associated with chronic oxidative and inflammatory stress linked to the multidrug resistance phenotype.

Estradiol, the primary estrogen, significantly influences breast cancer cell proliferation by activating estrogen receptor-related signaling pathways, which, when bound to E2, activate genes responsible for cell growth and division. Studies investigating the effect of E2 on the MCF-7 cell line are crucial for understanding hormonal mechanisms in breast cancer, which informed our research [31]. Interestingly, the response to 4-OHE2 differed from the action of E2 and 2-MeOE2. MCF-7/DOX cells were more sensitive to this catechol estrogen metabolite than MCF-7 cells, suggesting that 4-OHE2 may selectively target pathways altered during the development of chemoresistance. This may be related to increased ROS generation and DNA damage by 4-OHE2-derived quinones, which can promote oxidative and inflammation-associated stress, particularly in resistant cells that may lack sufficient DNA repair capacity under conditions of elevated oxidative stress [32,33].

### 3.2. Interactions Between Estrogens and Cadmium: Antagonism and Synergy

The interaction studies revealed a complex, concentration-dependent pattern of cytotoxic effects. Notably, E2 displayed both antagonistic and synergistic interactions with Cd, depending on the concentration. At low concentrations, E2 appeared to exert a protective effect against cadmium-induced toxicity, possibly via ER-mediated activation of antioxidant and cytoprotective pathways that may also attenuate inflammatory signaling and anti-apoptotic pathways. However, at intermediate concentrations (1–10 µM), a synergistic increase in cytotoxicity was observed, especially in MCF-7 cells, suggesting that E2 may also enhance Cd toxicity through an additive effect [34,35].

In contrast, 2-MeOE2 consistently exerted antagonistic effects when combined with cadmium, in both MCF-7 and MCF-7/DOX lines. This aligns with its known anti-angiogenic, pro-apoptotic, and ROS-scavenging properties, which may mitigate cadmium-induced oxidative damage [36]. These findings suggest that 2-MeOE2, as a non-classical estrogen metabolite, may have some therapeutic potential in cancers associated with oxidative stress. Our earlier research by Sawicka et al. [37] demonstrated that 2-MeOE2, in combination with cadmium, exhibited antagonistic activity in the SKOV-3 cell line (ovarian cancer), suggesting a similar mechanism in MCF-7 cells. Furthermore, 2-MeOE2 exhibits anti-inflammatory and antioxidant properties that may mitigate cadmium-induced oxidative damage [36]. Another study, although focused on neuroblastoma cells, demonstrated that 2-MeOE2 induces apoptosis through the production of ROS, which may be relevant in the context of its interaction with cadmium, which also generates ROS [38].

Our novel study also aimed to determine the effect of a metabolite with potentially carcinogenic properties, i.e., 4-OHE2, which exhibited a more heterogeneous interaction profile—predominantly antagonistic effects—except in the high-concentration combination in MCF-7/DOX cells, in which synergistic cytotoxicity was observed. The enhanced effect at 50 μM suggests that, at sufficient concentrations, 4-OHE2 may potentiate cadmium-induced oxidative and inflammation-associated stress.

Summarizing the results presented in Table 2, we noted that in the MCF-7 cell line in the presence of E2, antagonistic interactions dominated, particularly at lower concentrations (0.01–0.1 µM) of both compounds. However, synergistic effects were observed at higher concentrations: E2 at 1–10 µM in combination with both 1 µM and 50 µM CdCl_2_ led to a notable decrease in cell viability (down to 37%), indicating enhanced cytotoxicity. Interestingly, at a concentration of 50 µM E2 combined with 50 µM CdCl_2_ the effect returned to antagonistic, suggesting that there may be a concentration threshold above which cytoprotective mechanisms can be activated, which may be an interesting and important observation from our study.

Across all tested concentrations, the combination of 2-MeOE2 with CdCl_2_ consistently resulted in antagonistic effects. Even at higher concentrations (10–50 µM), cell viability remained relatively high (45–79%), suggesting that 2-MeOE2 may counteract Cd-induced cytotoxicity. No synergism was observed under any condition, highlighting the potential protective or neutral role of 2-MeOE2 in co-exposure scenarios, which is also an interesting conclusion.

In the case of 4-OHE2, the majority of combinations produced antagonistic effects, except at the highest concentration (50 µM) combined with 1 µM CdCl_2_, where a synergistic decrease in viability (35%) was observed. This result suggests that only high concentrations of catechol estrogens in combination with low-concentration cadmium may contribute to enhanced cytotoxicity in MCF-7 cells.

Summarizing the results presented in Table 3, we observed that in the MCF-7/DOX cell line the combination of E2 with CdCl_2_ resulted predominantly in antagonistic interactions, especially at higher estrogen concentrations. At low E2 levels (0.1–1 µM) and 1 µM CdCl_2_, synergistic effects were observed (viability 73–76%), indicating enhanced cytotoxicity compared to single-agent exposure. However, the same estrogen concentrations with 50 µM CdCl_2_ showed antagonistic effects, suggesting a shift in the nature of the interaction depending on the cadmium concentration. At higher E2 concentrations (10–50 µM), antagonism prevailed at both CdCl_2_ concentrations, with only a modest reduction in viability (65–52%). These findings point to a concentration-dependent modulation of cytotoxicity, with low-concentration E2 sensitizing MCF-7/DOX cells to low-concentration cadmium, but higher concentrations leading to potential adaptive responses that maintain cell viability, which is also a very interesting observation.

The combination of 2-MeOE2 with cadmium exhibited consistently antagonistic effects across all concentrations tested. Cell viability remained relatively high (80–89% with 1 µM CdCl_2_ and 54–75% with 50 µM CdCl_2_). No synergism was observed under any condition. This pattern indicates that 2-MeOE2 does not enhance the cytotoxic effect of cadmium in MCF-7/DOX cells, and may even exert a mild protective effect, possibly through antioxidant or cytostatic mechanisms.

Among the tested estrogens, 4-OHE2 produced the most pronounced synergistic effects, but only at the highest concentration. At 50 µM 4-OHE2, a marked synergistic interaction was observed with both 1 µM and 50 µM CdCl_2_, reducing viability to 63% and 12%, respectively. At lower concentrations (0.01–10 µM), however, all interactions remained antagonistic, with cell viability staying within the 60–100% range. This suggests that high concentrations of 4-OHE2, in combination with cadmium, may induce significant cytotoxicity in chemoresistant cells, likely via mechanisms involving oxidative stress or DNA damage, as 4-OHE2 is known to form genotoxic estrogen metabolites.

### 3.3. SOD1 Activation Highlights Redox Adaptation in Resistant Cells-: Comparison of Quantitative and Immunoenzymatic Approaches

Taking into account the quantitative determination of SOD1 activity, it was demonstrated that exposure to CdCl_2_ and estrogenic compounds markedly affects the enzymatic antioxidant response in both MCF-7 and MCF-7/DOX. The spectrophotometric RANSOD assay provided clear evidence of a concentration-dependent modulation of SOD1 activity following exposure to all tested agents, both as single compounds and in combined treatment. In particular, CdCl_2_ and catechol estrogens—4-OHE2 induced a pronounced increase in enzymatic activity, which was further enhanced under co-exposure conditions. This quantitative upregulation of SOD1 reflects a strong activation of the cellular antioxidant defense system in response to elevated oxidative stress. Importantly, the quantitative assay revealed consistent differences between the two cell models, with MCF-7/DOX cells exhibiting significantly higher basal SOD1 activity and a more pronounced inducible response compared with parental MCF-7 cells. This suggests that long-term adaptation to chemotherapeutic stress is associated with a reinforced enzymatic antioxidant capacity, which may contribute to the survival advantage of resistant cells under additional oxidative insults. Superoxide dismutase (SOD) is a central component of the antioxidant defense system, catalyzing the conversion of superoxide anion radicals (O_2_•^−^) into hydrogen peroxide (H_2_O_2_). In the context of cancer biology, including breast cancer, SOD1 plays a dual and highly context-dependent role [39]. While it protects biomolecules from oxidative damage, its sustained activation may also promote cancer cell survival under chronic oxidative and inflammatory conditions [40]. Therefore, SOD1 assessment after exposure to estrogens and cadmium provides insight primarily into redox adaptation mechanisms rather than direct functional causality. Consistent with this concept, the observed quantitative increases in SOD1 activity indicate that both CdCl_2_ and estrogenic compounds act as potent modulators of intracellular redox homeostasis, likely through enhanced ROS generation and subsequent activation of antioxidant defense pathways [41]. The strongest effects observed for 4-OHE2 suggest a particularly high pro-oxidant potential of catechol estrogen metabolites, which may undergo redox cycling and amplify oxidative stress signals [42]. In parallel, immunocytochemical analysis of SOD1 expression further confirmed elevated basal antioxidant capacity in MCF-7/DOX cells compared with MCF-7 cells. Upon exposure to CdCl_2_ and estrogens, either individually or in combination, MCF-7/DOX cells showed stronger SOD1 induction, particularly in response to 4-OHE2 and 2-MeOE2. This enhanced response likely reflects an adaptive increase in antioxidant defense mechanisms, contributing to reduced susceptibility to oxidative and inflammatory stress. Interestingly, 2-MeOE2 induced strong SOD1 upregulation without a proportional increase in cytotoxicity, suggesting a predominant role in redox modulation rather than direct cytotoxic action. In contrast, 4-OHE2 triggered both marked SOD1 induction and pronounced cytotoxicity at higher concentrations, indicating that excessive ROS generation may exceed the cellular antioxidant capacity. Overall, both quantitative and immunocytochemical analyses consistently demonstrated higher SOD1 levels in MCF-7/DOX cells across all treatments and concentrations, reflecting altered redox homeostasis and enhanced adaptive capacity to oxidative stress. The most pronounced differences between cell lines were observed following exposure to cadmium and higher concentrations of estrogenic compounds.

### 3.4. GST Activity Indicates Enhanced Detoxification Capacity in Chemoresistant Cells

Quantitative assessment of GST activity revealed marked differences between MCF-7 and MCF-7/DOX cells, both under basal conditions and following exposure to cadmium and estrogenic compounds. Consistent with the SOD1 results, MCF-7/DOX cells exhibited higher constitutive GST activity than parental MCF-7 cells, suggesting a more efficient detoxification system associated with the multidrug-resistant phenotype. Exposure to CdCl_2_ induced a concentration-dependent increase in GST activity in both cell lines, confirming the activation of glutathione-dependent defense mechanisms in response to xenobiotic-induced oxidative stress [43]. Among the tested estrogenic compounds, 4-OHE2 and 2-MeOE2 produced the strongest stimulation of GST activity, whereas E2 induced a comparatively weaker response. The pronounced effect of 4-OHE2 may be related to its ability to undergo redox cycling and generate reactive intermediates requiring detoxification through glutathione-dependent pathways [33]. Combined exposure to cadmium and estrogens resulted in a further increase in GST activity, particularly in MCF-7/DOX cells, indicating additive or complementary activation of cellular defense systems. The highest GST activity was observed following co-exposure to CdCl_2_ and 4-OHE2, supporting the hypothesis that simultaneous exposure to metalloestrogens and estrogen metabolites imposes a substantial oxidative and detoxification burden on breast cancer cells. The quantitative GST data suggest that chemoresistant MCF-7/DOX cells possess a greater capacity to activate glutathione-dependent detoxification pathways than hormone-sensitive MCF-7 cells. This enhanced GST response may contribute to the increased tolerance of resistant cells to oxidative stress and xenobiotic exposure, similarly to the elevated SOD1 activity observed in the present study [44].

Overall, GST activity followed the order 4-OHE2 > 2-MeOE2 > E2 in both cell lines, suggesting that estrogen metabolites, particularly the catechol estrogen 4-OHE2, exert stronger stimulatory effects on glutathione-dependent detoxification pathways than the parent hormone (Table 12).

### 3.5. GST-pi Expression Reflects Detoxification Capacity and Hormonal Regulation

Expression of GST-pi, a phase II detoxification enzyme involved in xenobiotic metabolism and a marker of drug resistance, was also significantly higher in MCF-7/DOX cells at baseline and after cadmium exposure. This is consistent with literature describing GST-pi as a factor contributing to the multidrug-resistant phenotype by facilitating the conjugation and elimination of toxic compounds and mitigating oxidative stress. The pi isoform of glutathione S-transferase was chosen for analysis because it is the major GST isoform in breast cancer cells and is closely associated with drug resistance, particularly to doxorubicin. Although other GST isoforms (e.g., alpha) are expressed in different tissues, GST-pi is the most relevant for evaluating cellular detoxification responses in the context of breast cancer and xenobiotic exposure [20,45,46].

Estrogens differentially changed GST-pi expression. E2 and 2-MeOE2 induced strong GST-pi expression in MCF-7 cells, consistent with hormone-sensitive regulation via estrogens [47]. However, in MCF-7/DOX cells, this effect was attenuated, likely related to the chemoresistance of cells. The relatively weak response of GST-pi to 4-OHE2, despite its genotoxic potential, suggests that its metabolism may rely on alternative detoxification pathways, independent of GSH. Nevertheless, combined exposure of 4-OHE2 with cadmium led to a significant increase in GST-pi expression in MCF-7/DOX cells, suggesting enhanced activation of stress and inflammation-related response pathways, although it does not demonstrate a direct role of GST-pi in modulating the effects of these compounds. Overall, MCF-7/DOX cells displayed higher GST-pi expression than MCF-7 in response to CdCl_2_, highlighting the heightened detoxification capacity of chemoresistant cells. In contrast, MCF-7 cells responded more strongly to estrogenic compounds, particularly E2 and 2-MeOE2, suggesting a greater sensitivity to hormone-mediated regulation. This differential pattern of GST-pi expression may reflect significant phenotypic differences between hormone-sensitive and drug-resistant breast cancer cells, which is relevant for understanding resistance mechanisms linked to redox balance and inflammation-associated pathways [48,49]. The results obtained in the present study describe differences between the cell lines, but do not provide evidence that GST-pi directly influences the cellular response to estrogens or cadmium.

It should be noted that the immunocytochemical analysis performed in this study provides qualitative and semi-quantitative information on SOD1 and GST-pi expression patterns. Although this method enables visualization of protein localization and comparison between cellular phenotypes, it primarily allows the assessment of relative differences in expression under the experimental conditions. At the same time, the manuscript has been supplemented with a quantitative assessment of SOD1 activity in the cell lines, enabling a more precise evaluation of changes in the antioxidant system in response to the experimental factors applied.

Taken together, the IC_50_ and CI data indicate that estrogen metabolites may modulate the cellular response to CdCl_2_ in a manner dependent on the type of metabolite, its concentration, and the cellular profile. These findings highlight the context-dependent nature of interactions between estrogens and cadmium. The analysis of SOD1, including both immunocytochemical assessment and quantitative measurement of enzymatic activity, together with the evaluation of GST-pi expression, indicates a significant involvement of the antioxidant system in the observed cellular responses.

In summary, the observed reduction in cell viability following the treatments is consistent with the aim of the study, as cytotoxicity represents a direct consequence of disturbances in oxidative-redox balance and alterations in detoxification mechanisms induced by cadmium and estrogen metabolites in breast cancer cells. Both cadmium and certain estrogen metabolites, particularly 4-hydroxyestradiol, are known to generate reactive oxygen species and induce oxidative stress, which may lead to cellular damage and decreased cell survival. Importantly, the assessment of the cytotoxicity of the tested compounds in both cell lines also enabled the determination of the type of estrogen–cadmium interaction, which constituted the main objective of our study.

## 4. Materials and Methods

### 4.1. Materials

Dulbecco’s Modified Eagle’s Medium (DMEM, high-glucose), phosphate-buffered saline (PBS), fetal bovine serum (FBS), and penicillin-streptomycin solution were purchased from Biological Industries (Beit HaEmek, Israel). Cadmium chloride (CdCl_2_), dimethyl sulfoxide (DMSO), deionized water, ethanol (96%), 17β-estradiol (E2), 2-methoxyestradiol (2-MeOE2), and 4-hydroxyestradiol (4-OHE2) were obtained from Sigma-Aldrich (Burlington, MA, USA). TrypLE™ Express enzyme was purchased from Gibco (Waltham, MA, USA). For immunocytochemical analysis, the Expose™ Mouse and Rabbit Specific HRP/DAB Detection IHC Kit (ab80436, Abcam, Waltham, MA, USA) was used. Primary antibodies included anti-SOD1 rabbit polyclonal antibody (orb39428, Biorbyt, Cambridge, UK) and anti-GSTπ rabbit polyclonal antibody (orb678653, Biorbyt, Cambridge, UK). The list of tested compounds and their chemical structures is presented in Table 13.

### 4.2. Cell Culture

The human estrogen-dependent breast adenocarcinoma cell line MCF-7 was obtained from CLS Cell Lines Service GmbH (Eppelheim, Germany). The doxorubicin-resistant subline MCF-7/DOX was established by continuous culture of MCF-7 cells in the presence of low concentrations of doxorubicin over a period of 3 months. The resistance phenotype of MCF-7/DX cells is based on previously reported literature, while our study focuses on evaluating cytotoxicity and immunocytochemical analysis of SOD1 and GSTπ in these cells. Both cell lines were maintained at 37 °C in a humidified atmosphere containing 5% CO_2_ using a Steri-Cult^®^ Automated CO_2_ Incubator (Thermo Scientific, distributed by ALAB, Warsaw, Poland). Cells were cultured in DMEM high-glucose medium (4500 mg/L; supplemented with 10% fetal bovine serum and 1% penicillin-streptomycin (10,000 U/mL penicillin and 10 mg/mL streptomycin from Biological Industries (Beit HaEmek, Israel).

### 4.3. Tested Compounds

The tested compounds included estrogens (E2, 2-MeOE2, 4-OHE2) and CdCl_2_ as a source of cadmium ions. Stock solutions of estrogens were prepared in 96% ethanol at a concentration of 10 mM, while cadmium chloride was dissolved in sterile deionized water to obtain a 10 mM solution. Working concentrations used in experiments were as follows: estrogens: 0.01, 0.1, 1.0, 10.0, and 50.0 µM; CdCl_2_: 0.1, 1.0, 5.0, 10.0, and 50.0 µM.

### 4.4. Cytotoxicity Assay of Individual Estrogens and CdCl_2_

The cytotoxicity of the tested compounds was assessed using the MTT assay, following the manufacturer’s protocol (Sigma-Aldrich, Poznań, Poland). Cells were seeded into 96-well plates (Nunc, Nunclon™ Surface, Biokom, Janki, Poland) at a density of 5 × 10^4^ cells/well in DMEM. After 24 h of incubation at 37 °C in 5% CO_2_ to allow cell adhesion, the culture medium was replaced with 200 µL of medium containing the test compounds. Each estrogen (E2, 2-MeOE2, 4-OHE2) and CdCl_2_ was tested individually at the concentrations listed in Section 4.3. Cells were incubated with the compounds for 48 h. A negative control consisting of cells incubated in complete culture medium without any compounds was included in each experiment. All conditions were tested in triplicate. Following exposure, 20 µL of MTT solution (5 mg/mL) was added to each well to obtain a final concentration of 0.5 mg/mL. Plates were incubated for 4 h at 37 °C. Subsequently, the medium was removed, and formazan crystals formed in viable cells were solubilized using DMSO. Absorbance was measured at 570 nm using a microplate reader. Results were expressed as a percentage of cell viability compared to the untreated control.

### 4.5. Combined Effect of Estrogens and Cadmium on Cell Viability

To evaluate potential interactive effects, a simultaneous exposure model was employed. In this model, cells were treated concurrently with cadmium chloride and one of the tested estrogens. Concentrations for the combined treatment were selected based on preliminary cytotoxicity assays performed on MCF-7 and MCF-7/DOX breast cancer cells, ensuring inclusion of both sub-cytotoxic and cytotoxic ranges. In addition, previously published studies in hormone-dependent cancer cell lines, including ovarian cancer models, were considered to define biologically relevant concentration ranges. The following concentration ranges were applied: estrogens (E2, 2-MeOE2, 4-OHE2): 0.01, 0.1, 1.0, 10.0, 50.0 µM and CdCl_2_: 1.0 and 50.0 µM. Cells were incubated with the mixtures for 48 h under standard culture conditions (37 °C, 5% CO_2_). Following exposure, cytotoxicity was determined using the MTT assay as described in Section 4.4. The results were analyzed to evaluate potential synergistic or antagonistic effects between cadmium and estrogens.

### 4.6. Combination Index

To estimate the type of interaction, the combination index (CI) was calculated using CompuSyn software (Version 1.0) The results were interpreted as follows: CI = 1 indicates the additive effect of the test substances on the viability of breast cancer cells, CI < 1 indicates synergy (S) between the compounds used for the study, while CI > 1 indicates the antagonism (A) that occurs between them [50].

### 4.7. Immunocytochemical Analysis of SOD1 Expression in MCF-7 and MCF-7/DOX Breast Cancer Cells

To assess potential interactions of the tested compounds with respect to both enzymes, SOD and GST-pi, concentrations of 0.1 μM and 10 μM for estrogens were selected, as well as the same concentration range for cadmium, i.e., 0.1 μM and 10 μM.

The expression of superoxide dismutase 1 (SOD1) was evaluated in human breast cancer cell lines MCF-7 and MCF-7/DOX (doxorubicin-resistant) following exposure to individual compounds and mixtures of estrogens and Cd, in accordance with the viability assay conditions. Cells were seeded onto 10-well microscope slides (Thermo Scientific, Waltham, MA, USA) and incubated for 24 h. Following incubation, the slides were rinsed with phosphate-buffered saline (PBS) and fixed with 4% paraformaldehyde. Immunocytochemical staining was then performed using the Expose Mouse and Rabbit Specific HRP/DAB Detection IHC Kit (Abcam, Waltham, MA, USA; ab80436), which included the following components: Mouse Determination Reagent, HRP Conjugate, DAB Substrate, DAB Chromogen, and Hydrogen Peroxide Block. After three washes with PBS (3 × 5 min), endogenous peroxidase activity was quenched with 1% hydrogen peroxide for 30 min. Cells were then permeabilized with 1% Triton X-100 (Merck, Poland–Sigma-Aldrich) in PBS (LabEmpire, Rzeszów, Poland). Next, the cells were incubated overnight at 4 °C with a primary antibody: anti-SOD1 rabbit polyclonal antibody (orb39428, Biorbyt, Cambridge, UK). This was followed by incubation with an HRP-conjugated secondary antibody, according to the manufacturer’s protocol. Visualization of the signal was achieved using a DAB/H_2_O_2_ chromogenic reaction, and slides were counterstained with hematoxylin (Roth, Poland) for 3 min. Subsequently, samples were dehydrated through an ethanol gradient (Chempur, Rzeszów, Poland), cleared in xylene (Chempur, Piekary Śląskie, Poland), and mounted using DPX mounting medium (Aqua-Med ZpamKolasa, Łódź, Poland). Images were acquired using a vertical microscope (Olympus BX53, Warsaw, Poland). Staining intensity was categorized as follows: (–) negative (no staining), (+) weak, (++) moderate, and (+++) strong. Qualitative data were summarized in tabular form (Table 5 and Table 6).

### 4.8. Immunocytochemical Analysis of GST-pi Expression in MCF-7 and MCF-7/DOX Breast Cancer Cells

The expression of glutathione S-transferase pi (GSTπ) was evaluated in human breast cancer cell lines MCF-7 and MCF-7/DOX following exposure to individual compounds and mixtures of estrogens and CdCl2. MCF-7 and MCF-7/DOX cells were seeded onto microscope slides and incubated for 24 h, followed by treatment with test compounds. After incubation, cells were fixed with 4% paraformaldehyde, rinsed with PBS, and stained using the Expose HRP/DAB Detection Kit (Abcam). Endogenous peroxidase activity was blocked with 1% H_2_O_2_, and cells were permeabilized with 1% Triton X-100. Slides were incubated overnight at 4 °C with the primary antibody: anti-GSTπ rabbit polyclonal antibody (orb101235, Biorbyt, Cambridge, UK). An HRP-conjugated secondary antibody was then applied, and the signal was developed with DAB. Nuclei were counterstained with hematoxylin, and slides were dehydrated, cleared in xylene, and mounted in DPX. Images were acquired using an Olympus BX53 microscope. Staining intensity was classified as follows: (–) negative, (+) weak, (++) moderate, (+++) strong. The intensity of immunoreactivity was summarized in Table 7 and Table 8.

### 4.9. Quantitative Determination of SOD1 Activity Following Exposure to Single Compounds or in Combined Treatment in MCF-7 and MCF-7/DOX Cell Lines Using the RANSOD Spectrophotometric Assay in the Breast Cancer Cell Line

The activity of superoxide dismutase 1 (SOD1, Cu/Zn-SOD) was determined in lysates obtained from MCF-7 and MCF-7/DOX cells exposed to estrogenic compounds (E2, 2-MeOE2, and 4-OHE2) and cadmium chloride (CdCl_2_), either as single agents or in combined treatment. SOD1 activity was measured using a spectrophotometric assay with the RANSOD kit (Randox Laboratories), according to the manufacturer’s instructions. The assay is based on the ability of SOD to inhibit the reduction of 2-(4-iodophenyl)-3-(4-nitrophenol)-5-phenyltetrazolium chloride (I.N.T.) by superoxide radicals generated in the xanthine–xanthine oxidase system.

Cells were cultured under standard conditions (37 °C, humidified atmosphere with 5% CO_2_). After treatment completion, cells were washed twice with ice-cold PBS, detached, and centrifuged at 1500× *g* for 5 min at 4 °C. Cell pellets were lysed in ice-cold phosphate buffer (50 mM, pH 7.4) containing 0.1 mM EDTA. Lysates were homogenized and centrifuged at 10,000× *g* for 15 min at 4 °C to remove cellular debris, and the resulting supernatants were collected for analysis. SOD1 activity was quantified by measuring the inhibition of I.N.T. reduction to red formazan. Absorbance changes were recorded spectrophotometrically at 505 nm. One unit of SOD activity was defined as the amount of enzyme required to cause 50% inhibition of I.N.T. reduction under assay conditions. Results were expressed as units per milligram of total protein (U/mg protein). Protein concentration was measured using the Pierce™ BCA Protein Assay Kit according to the manufacturer’s instructions (Table 9 and Table 10).

### 4.10. Quantitative Determination of GST Activity Following Exposure to Single Compounds or Combined Treatment in MCF-7 and MCF-7/DOX Breast Cancer Cell Lines Using the CDNB Spectrophotometric Assay

Glutathione S-transferase (GST) activity was determined in cell lysates obtained from MCF-7 and MCF-7/DOX cells following 48 h exposure to cadmium chloride, E2, 4-OHE2, 2-MeOE2, and their respective combinations. After treatment, the cells were washed twice with ice-cold phosphate-buffered saline (PBS), harvested, and processed for enzymatic analysis. Approximately 1 × 10^6^ cells were used for each sample. Cells were lysed under ice-cold conditions and centrifuged at 10,000× *g* for 15 min at 4 °C. The resulting supernatants were collected and used for GST activity determination. Protein concentration was measured using the Pierce™ BCA Protein Assay Kit according to the manufacturer’s instructions. GST activity was determined according to the method of Habig et al. [51], based on the conjugation of reduced glutathione (GSH) with 1-chloro-2,4-dinitrobenzene (CDNB). The assay was performed in clear 96-well microplates using a Synergy™ microplate reader (BioTek Instruments, Winooski, VT, USA). The reaction mixture consisted of 100 mM potassium phosphate buffer (pH 6.5), 1 mM reduced glutathione (GSH), and 1 mM CDNB. Briefly, 20 µL of cell lysate was added to 180 µL of reaction mixture, resulting in a final reaction volume of 200 µL per well. Blank samples containing all assay components except the cell lysate were included to correct for non-enzymatic conjugate formation. The reaction was initiated by the addition of CDNB, and the increase in absorbance was monitored at 340 nm for 5 min. GST activity was calculated from the linear portion of the absorbance curve after subtraction of blank values. The rate of formation of the CDNB–GSH conjugate was calculated using the molar extinction coefficient ε = 9.6 mM^−1^ cm^−1^. GST activity was expressed as nanomoles of CDNB–GSH conjugate formed per minute per milligram of protein (nmol/min/mg protein). Each sample was analyzed in triplicate. The results were calculated per mg of protein content and expressed as mean ± standard deviation (SD).

### 4.11. Statistical Analysis

All values were expressed as mean ± SD. Differences between all groups were assessed using one-way analysis of variance (ANOVA), which compares three or more unmatched groups based on the assumption that the populations are Gaussian. When ANOVA indicated significant differences, Tukey’s post hoc test was applied to determine intergroup differences. All analyses were performed using GraphPad Prism 7 software (GraphPad Software, San Diego, CA, USA). Values of *p* < 0.05 were considered statistically significant. The concentration–effect analysis of the combination treatment was performed using the combination index (CI) method implemented in CompuSyn software (Version 1.0).

## 5. Conclusions

The presented results provide descriptive insights into the effects of environmental metalloestrogens in combination with endogenous estrogens and their metabolites on oxidative and redox-regulated responses in breast cancer cells. Cadmium, as an environmental toxin with known pro-inflammatory properties, influenced both cell viability and redox balance, with these effects varying depending on the specific estrogen compound and the cell line studied. Differences between MCF-7 and MCF-7/DOX cells likely reflect distinct properties in redox homeostasis, hormonal sensitivity, and detoxification capacity, as well as adaptive responses to chronic oxidative stress, which may contribute to their differential responses to xenobiotics. The observed increase in activity and expression of SOD1, GST and GST-pi in MCF-7/DOX cells indicates enhanced antioxidant and detoxification responses, functionally linked to the regulation of oxidative stress and redox adaptive signaling pathways. However, further functional studies will be necessary to clarify the specific roles of the examined compounds in redox-regulated cellular responses. From a therapeutic perspective, the protective effect of 2-MeOE2, potentially related to its modulation of redox homeostatic responses, and the selective susceptibility of resistant cells to 4-OHE2–CdCl_2_ combinations associated with enhanced oxidative and inflammatory stress, may provide a rationale for the development of new strategies for breast cancer treatment. Importantly, our findings suggest a specific interaction between estrogenic compounds and cadmium, which was predominantly antagonistic, whereas synergistic effects were observed less frequently. Overall, our study underscores the importance of concentration-dependent interactions between environmental metals and pathways regulated by 17β-estradiol and its metabolites, particularly in the context of cytotoxicity and redox imbalance relevant to breast cancer progression and therapy resistance.

## Figures and Tables

**Table 1:** IC50 for individual compounds after simultaneous effect on MCF-7 and MCF-7/DOX, measured by MTT (expressed in µM).

Cell Line	IC 50 for Examined Compounds [µM]			
	CdCl_2_	E2	2-MeOE2	4-OHE2
MCF-7	4.26	21.24	35.21	16.92
MCF-7/DOX	9.74	35.23	>50.00	6.74

**Table 2:** The type of interaction after the combined effect of CdCl_2_ and estrogens on MCF-7 viability (MTT—48 h incubation) using the CompuSyn program; A—antagonism, S—synergism, CI—combination index.

Estrogen		1 µM CdCl_2_			50 µM CdCl_2_		
	C [μM]	Viability [%]	CI	Effect	Viability [%]	CI	Effect
E_2_	0.01	88	268.21	A	89	321.23	A
	0.1	65	8.32	A	59	19.84	A
	1	70	112.34	A	41	0.45	S
	10	58	0.724	S	37	0.216	S
	50	83	152.34	A	30	2.85	A
2-MeOE2	0.01	100	275.31	A	70	120.43	A
	0.1	100	190.45	A	67	85.32	A
	1	107	320.34	A	48	20.35	A
	10	79	18.67	A	48	19.34	A
	50	60	16.43	A	45	9.43	A
4-OHE2	0.01	88	153.21	A	70	92.67	A
	0.1	65	29.13	A	52	5.97	A
	1	47	10.5	A	47	4.39	A
	10	37	7.23	A	37	6.21	A
	50	35	0.375	S	35	7.88	A

**Table 3:** The type of interaction after the combined effect of CdCl_2_ and estrogens on MCF-7/DOX viability (MTT—48 h incubation) using the CompuSyn program; A—antagonism, S—synergism, CI—combination index.

Estrogen		1 µM CdCl_2_			50 µM CdCl_2_		
	C [μM]	Viability [%]	CI	Effect	Viability [%]	CI	Effect
E_2_	0.01	97	458.21	A	75	5.97	A
	0.1	76	0.82	S	74	8.37	A
	1	73	0.72	S	70	2.58	A
	10	75	2.80	A	69	3.12	A
	50	65	3.21	A	52	2.83	A
2-MeOE2	0.01	89	286.43	A	72	7.34	A
	0.1	86	192.36	A	75	8.19	A
	1	80	73.89	A	73	3.25	A
	10	84	170.28	A	62	2.18	A
	50	68	2.19	A	54	1.19	A
4-OHE2	0.01	95	518.98	A	80	71.35	A
	0.1	96	492.43	A	82	114.74	A
	1	100	741.97	A	70	5.12	A
	10	99	625.84	A	60	2.13	A
	50	63	0.85	S	12	0.25	S

**Table 4:** IC50 for the mixture of compounds after the combined effect on MCF-7 and MCF-7/DOX, measured by MTT (expressed in µM).

Cell Line	IC 50 [µM] for Examined Mixture of Compounds [µM]						
	CdCl_2_	CdCl_2_ (1 µM) + E2	CdCl_2_ (50 µM) + E2	CdCl_2_ (1 µM) + 2-MeOE2	CdCl_2_ (50 µM) + 2-MeOE2	CdCl_2_ (1 µM) + 4-OHE2	CdCl_2_ (50 µM) + 4-OHE2
MCF-7	4.26	>50.00	1.04	>50.00	34.94	1.76	0.55
MCF-7/DOX	9.74	29.23	32.52	>50.00	38.35	>50.00	4.06

**Table 5:** Semi-quantitative grading of SOD1 immunocytochemical staining in MCF-7 and MCF-7/DOX cell lines following treatment with individual compounds. The staining intensity was evaluated as follows: (+) weak, (++) moderate, and (+++) strong positive.

Cell Line	Compound	Concentration [µM]	The Intensity of the Reaction
MCF-7	CdCl_2_ CdCl_2_	0.1 10.0	+ +/++
MCF-7/DOX	CdCl_2_ CdCl_2_	0.1 10.0	++ ++/+++
MCF-7	E2 E2	0.1 10.0	++ +
MCF-7/DOX	E2 E2	0.1 10.0	++ ++/+++
MCF-7	2-MeOE2 2-MeOE2	0.1 10.0	++ +
MCF-7/DOX	2-MeOE2 2-MeOE2	0.1 10.0	++ +
MCF-7	4-OHE2 4-OHE2	0.1 10.0	+/++ ++/+++
MCF-7/DOX	4-OHE2 4-OHE2	0.1 10.0	++ ++/+++
MCF-7 MCF-7/DOX	Control Control	+ ++	+ ++

**Table 6:** Semi-quantitative evaluation of SOD1 immunocytochemical staining in MCF-7 and MCF-7/DOX cell lines following combined exposure to estrogens and cadmium chloride. The staining intensity was evaluated as follows: (+) weak, (++) moderate, and (+++) strong positive.

Cell Line	Estrogen [µM]	CdCl_2_ [µM]	The Intensity of the Reaction
MCF-7 MCF-7/DOX	E2 0.1	0.1 10.0 0.1 10.0	++ ++ +++ ++/+++
MCF-7 MCF-7/DOX	E2 10.0	0.1 10.0 0.1 10.0	++ + ++ ++
MCF-7 MCF-7/DOX	2-MeOE2 0.1	0.1 10.0 0.1 10.0	+++ +++ ++ +++
MCF-7 MCF-7/DOX	2-MeOE2 10.0	0.1 10.0 0.1 10.0	++ ++ ++/+++ +++
MCF-7 MCF-7/DOX	4-OHE2 0.1	0.1 10.0 0.1 10.0	+/++ +++ ++/+++ +++
MCF-7 MCF-7/DOX	4-OHE2 10.0	0.1 10.0 0.1 10.0	+/++ +/++ ++ ++/+++
MCF-7 MCF-7/DOX	Control Control		+ ++

**Table 7:** Semi-quantitative grading of GST-pi immunocytochemical staining in MCF-7 and MCF-7/DOX cell lines after treatment with individual compounds. The staining intensity was evaluated as follows: (+) weak, (++) moderate, and (+++) strong positive.

Cell Line	Compound	Concentration [µM]	The Intensity of the Reaction
MCF-7	CdCl_2_ CdCl_2_	0.1 10.0	+ ++/++
MCF-7/DOX	CdCl_2_ CdCl_2_	0.1 10.0	++ ++/+++
MCF-7	E2 E2	0.1 10.0	+/++ +++
MCF-7/DOX	E2 E2	0.1 10.0	++ +/++
MCF-7	2-MeOE2 2-MeOE2	0.1 10.0	++ ++/+++
MCF-7/DOX	2-MeOE2 2-MeOE2	0.1 10.0	+/++ +/++
MCF-7	4-OHE2 4-OHE2	0.1 10.0	+/++ +/++
MCF-7/DOX	4-OHE2 4-OHE2	0.1 10.0	++ ++
MCF-7 MCF-7/DOX	Control Control		+ +

**Table 8:** Semi-quantitative evaluation of GST-pi immunocytochemical staining in MCF-7 and MCF-7/DOX cell lines following combined exposure to oestrogens and cadmium chloride. The staining intensity was evaluated as follows: (+) weak, (++) moderate, and (+++) strong positive.

Cell Line	Estrogen [µM]	CdCl_2_ [µM]	The Intensity of the Reaction
MCF-7 MCF-7/DOX	E2 0.1	0.1 10.0 0.1 10.0	++ ++ +++ ++
MCF-7 MCF-7/DOX	E2 10.0	0.1 10.0 0.1 10.0	+++ ++ ++ ++
MCF-7 MCF-7/DOX	2-MeOE2 0.1	0.1 10.0 0.1 10.0	++ ++ ++ ++/+++
MCF-7 MCF-7/DOX	2-MeOE2 10.0	0.1 10.0 0.1 10.0	++ ++ ++/+++ +++
MCF-7 MCF-7/DOX	4-OHE2 0.1	0.1 10.0 0.1 10.0	++ ++ +/++ ++
MCF-7 MCF-7/DOX	4-OHE2 10.0	0.1 10.0 0.1 10.0	+/++ ++ +++ ++/+++
MCF-7 MCF-7/DOX	Control Control		+ ++

**Table 9:** Activity of SOD1 in MCF-7 and MCF-7/DOX cells after single exposure to CdCl_2_ or estrogens (E2, 2-MeOE2, and 4-OHE2). Data are mean ± SD (n = 3). Statistical significance vs. corresponding control (* *p* < 0.05; ** *p* < 0.01; *** *p* < 0.001).

Cell Line	Compound	Concentration [µM]	SOD1 Activity [U/mg Protein]
MCF-7	Control	—	12.4 ± 0.8
MCF-7/DOX	Control	—	18.9 ± 1.1
MCF-7	CdCl_2_	0.1	14.1 ± 0.9 *
MCF-7	CdCl_2_	10.0	22.8 ± 1.4 ***
MCF-7/DOX	CdCl_2_	0.1	20.3 ± 1.2 *
MCF-7/DOX	CdCl_2_	10.0	31.7 ± 1.9 ***
MCF-7	E2	0.1	13.5 ± 0.7
MCF-7	E2	10.0	16.8 ± 1.0 **
MCF-7/DOX	E2	0.1	19.6 ± 1.0
MCF-7/DOX	E2	10.0	24.1 ± 1.5 **
MCF-7	2-MeOE2	0.1	15.9 ± 0.9 **
MCF-7	2-MeOE2	10.0	27.5 ± 1.8 ***
MCF-7/DOX	2-MeOE2	0.1	22.1 ± 1.3 **
MCF-7/DOX	2-MeOE2	10.0	35.9 ± 2.0 ***
MCF-7	4-OHE2	0.1	17.4 ± 1.0 **
MCF-7	4-OHE2	10.0	30.6 ± 2.1 ***
MCF-7/DOX	4-OHE2	0.1	24.8 ± 1.6 ***
MCF-7/DOX	4-OHE2	10.0	41.2 ± 2.5 ***

**Table 10:** Activity of SOD1 in MCF-7 and MCF-7/DOX cells after combined exposure to CdCl_2_ and estrogens (E2, 2-MeOE2, and 4-OHE2). Data are mean ± SD (n = 3). Statistical significance vs. corresponding control (* *p* < 0.05; ** *p* < 0.01; *** *p* < 0.001).

Cell Line	Estrogen	Estrogen [µM]	CdCl_2_ [µM]	SOD1 Activity [U/mg Protein]
MCF-7	Control	—	—	12.4 ± 0.8
MCF-7/DOX	Control	—	—	18.9 ± 1.1
MCF-7	E2	0.1	0.1	16.3 ± 1.0 *
MCF-7	E2	0.1	10.0	24.8 ± 1.5 ***
MCF-7/DOX	E2	0.1	0.1	22.4 ± 1.3 *
MCF-7/DOX	E2	0.1	10.0	34.9 ± 2.1 ***
MCF-7	E2	10.0	0.1	19.8 ± 1.2 **
MCF-7	E2	10.0	10.0	29.7 ± 1.9 ***
MCF-7/DOX	E2	10.0	0.1	27.5 ± 1.7 **
MCF-7/DOX	E2	10.0	10.0	39.6 ± 2.4 ***
MCF-7	2-MeOE2	0.1	0.1	18.6 ± 1.1 **
MCF-7	2-MeOE2	0.1	10.0	31.4 ± 2.0 ***
MCF-7/DOX	2-MeOE2	0.1	0.1	25.9 ± 1.5 **
MCF-7/DOX	2-MeOE2	0.1	10.0	42.1 ± 2.6 ***
MCF-7	2-MeOE2	10.0	0.1	24.7 ± 1.5 ***
MCF-7	2-MeOE2	10.0	10.0	38.8 ± 2.3 ***
MCF-7/DOX	2-MeOE2	10.0	0.1	34.8 ± 2.0 ***
MCF-7/DOX	2-MeOE2	10.0	10.0	49.3 ± 3.1 ***
MCF-7	4-OHE2	0.1	0.1	20.5 ± 1.3 **
MCF-7	4-OHE2	0.1	10.0	35.9 ± 2.2 ***
MCF-7/DOX	4-OHE2	0.1	0.1	29.8 ± 1.8 ***
MCF-7/DOX	4-OHE2	0.1	10.0	46.7 ± 2.8 ***
MCF-7	4-OHE2	10.0	0.1	27.6 ± 1.7 ***
MCF-7	4-OHE2	10.0	10.0	43.5 ± 2.6 ***
MCF-7/DOX	4-OHE2	10.0	0.1	38.9 ± 2.4 ***
MCF-7/DOX	4-OHE2	10.0	10.0	56.4 ± 3.5 ***

**Table 11:** Activity of GST in MCF-7 and MCF-7/DOX cells after single exposure to CdCl_2_ or estrogens (E2, 2-MeOE2, and 4-OHE2). Data are mean ± SD (n = 3). Statistical significance vs. corresponding control (* *p* < 0.05; ** *p* < 0.01; *** *p* < 0.001).

Cell Line	Compound	Concentration [µM]	GST Activity [nmol/min/mg Protein]
MCF-7	Control	—	42.8 ± 2.6
MCF-7/DOX	Control	—	68.5 ± 4.1
MCF-7	CdCl_2_	0.1	48.3 ± 3.0 *
MCF-7	CdCl_2_	10.0	76.9 ± 4.7 ***
MCF-7/DOX	CdCl_2_	0.1	75.2 ± 4.5 *
MCF-7/DOX	CdCl_2_	10.0	108.4 ± 6.2 ***
MCF-7	E2	0.1	45.1 ± 2.7
MCF-7	E2	10.0	55.9 ± 3.5 **
MCF-7/DOX	E2	0.1	71.8 ± 4.0
MCF-7/DOX	E2	10.0	84.3 ± 5.1 **
MCF-7	2-MeOE2	0.1	53.4 ± 3.1 **
MCF-7	2-MeOE2	10.0	89.2 ± 5.6 ***
MCF-7/DOX	2-MeOE2	0.1	82.6 ± 4.9 **
MCF-7/DOX	2-MeOE2	10.0	122.7 ± 7.1 ***
MCF-7	4-OHE2	0.1	60.7 ± 3.7 **
MCF-7	4-OHE2	10.0	101.4 ± 6.3 ***
MCF-7/DOX	4-OHE2	0.1	93.8 ± 5.6 ***
MCF-7/DOX	4-OHE2	10.0	139.6 ± 8.4 ***

**Table 12:** Activity of GST in MCF-7 and MCF-7/DOX cells after combined exposure to CdCl_2_ and estrogens (E2, 2-MeOE2, and 4-OHE2). Data are mean ± SD (n = 3). Statistical significance vs. corresponding control (* *p* < 0.05; ** *p* < 0.01; *** *p* < 0.001).

Cell Line	Estrogen	Estrogen [µM]	CdCl_2_ [µM]	GST Activity [nmol/min/mg Protein]
MCF-7	Control	—	—	42.8 ± 2.6
MCF-7/DOX	Control	—	—	68.5 ± 4.1
MCF-7	E2	0.1	0.1	52.4 ± 3.1 *
MCF-7	E2	0.1	10.0	82.7 ± 4.9 ***
MCF-7/DOX	E2	0.1	0.1	79.8 ± 4.8 *
MCF-7/DOX	E2	0.1	10.0	114.2 ± 6.8 ***
MCF-7	E2	10.0	0.1	61.5 ± 3.8 **
MCF-7	E2	10.0	10.0	95.4 ± 5.7 ***
MCF-7/DOX	E2	10.0	0.1	90.7 ± 5.4 **
MCF-7/DOX	E2	10.0	10.0	128.6 ± 7.5 ***
MCF-7	2-MeOE2	0.1	0.1	64.2 ± 3.9 **
MCF-7	2-MeOE2	0.1	10.0	101.8 ± 6.1 ***
MCF-7/DOX	2-MeOE2	0.1	0.1	95.4 ± 5.6 **
MCF-7/DOX	2-MeOE2	0.1	10.0	137.2 ± 8.0 ***
MCF-7	2-MeOE2	10.0	0.1	82.9 ± 4.9 ***
MCF-7	2-MeOE2	10.0	10.0	119.7 ± 7.1 ***
MCF-7/DOX	2-MeOE2	10.0	0.1	117.5 ± 6.9 ***
MCF-7/DOX	2-MeOE2	10.0	10.0	158.4 ± 9.3 ***
MCF-7	4-OHE2	0.1	0.1	72.6 ± 4.3 **
MCF-7	4-OHE2	0.1	10.0	114.8 ± 6.8 ***
MCF-7/DOX	4-OHE2	0.1	0.1	106.9 ± 6.3 ***
MCF-7/DOX	4-OHE2	0.1	10.0	149.5 ± 8.8 ***
MCF-7	4-OHE2	10.0	0.1	96.8 ± 5.8 ***
MCF-7	4-OHE2	10.0	10.0	136.2 ± 8.1 ***
MCF-7/DOX	4-OHE2	10.0	0.1	134.7 ± 7.9 ***
MCF-7/DOX	4-OHE2	10.0	10.0	182.9 ± 10.7 ***

**Table 13:** The list of compounds used in the experiments.

The Name of Compound and Manufacturer	The Structure
E2 SIGMA-ALRDICH	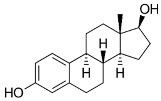
2-MeOE2 SIGMA-ALDRICH	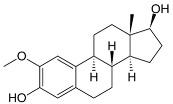
4-OHE2 SIGMA-ALDRICH	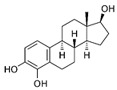
Cadmium chloride SIGMA-ALDRICH	CdCl_2_

## Data Availability

The data presented in this work is available in the article.
